# Conservation of ultrafast photoprotective mechanisms with increasing molecular complexity in sinapoyl malate derivatives

**DOI:** 10.1002/cphc.202000429

**Published:** 2020-08-04

**Authors:** Lewis A. Baker, Michael Staniforth, Amandine L. Flourat, Florent Allais, Vasilios G. Stavros

**Affiliations:** ^1^ Department of Chemistry University of Warwick Gibbet Hill Road Coventry CV4 7AL United Kingdom; ^2^ Faculty of Engineering and Physical Sciences University of Surrey 388 Stag Hill Guildford GU2 7XH United Kingdom; ^3^ URD Agro-Biotechnologies Industrielles (ABI), CEBB, AgroParisTech 51110 Pomacle France

**Keywords:** sinapoyl malate derivatives, steric hindrance, ultrafast photochemistry, plant sunscreens, solution phase

## Abstract

Sinapoyl malate is a natural plant sunscreen molecule which protects leaves from harmful ultraviolet radiation. Here, the ultrafast dynamics of three sinapoyl malate derivatives, sinapoyl L‐dimethyl malate, sinapoyl L‐diethyl malate and sinapoyl L‐di‐*t*‐butyl malate, have been studied using transient electronic absorption spectroscopy, in a dioxane and methanol solvent environment to investigate how well preserved these dynamics remain with increasing molecular complexity. In all cases it was found that, upon photoexcitation, deactivation occurs via a *trans‐cis* isomerisation pathway within ∼20–30 ps. This *cis*‐photoproduct, formed during photodeactivation, is stable and longed‐lived for all molecules in both solvents. The incredible levels of conservation of the isomerisation pathway with increased molecular complexity demonstrate the efficacy of these molecules as ultraviolet photoprotectors, even in strongly perturbing solvents. As such, we suggest these molecules might be well‐suited for augmentations to further improve their photoprotective efficacy or chemical compatibility with other components of sunscreen mixtures, whilst conserving their underlying photodynamic properties.

## Graphical abstract

UV‐visible pump‐probe spectroscopy is used to understand the photodeactivation mechanism in a series of sinapoyl malate derivatives. In sinapoyl malate, this photodeactivation occurs *via* a *trans*‐*cis* isomeration pathway within ∼20–30 ps. This pathway appears remarkably well‐conserved even in the most augmented system studied, sinapoyl L‐di‐*t*‐butyl malate. As such, these molecules might be well‐suited for further augmentation to improve their efficacy or chemical compatibility within sunscreen mixtures.

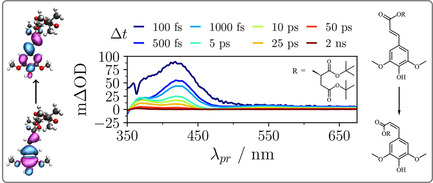

## 1 Introduction

Plants exhibit a burden of disease curve when it comes to ultraviolet (UV) light exposure.[^1^, ^2^] On the one hand, they require sunlight for photosynthesis which exposes them to UV radiation. They also require UV‐B radiation to act as a signal transducer to control a myriad of vital biochemical pathways.^[3]^ On the other hand, too much exposure to UV radiation can damage photosynthetic machinery and even make them more susceptible to invading pathogens.[^4^, ^5^] A method many plants use, such as the *Arabidopsis thaliana* species, is to synthesise and deposit UV absorbing metabolites in the upper epidermis of the their leaves.[^6^, ^7^] By increasing or decreasing the concentration of these metabolites in the leaves, the effective UV exposure reaching sensitive areas of the plant is controlled, thereby finding some equilibrium between too much, and too little UV exposure.

**Figure 1:**
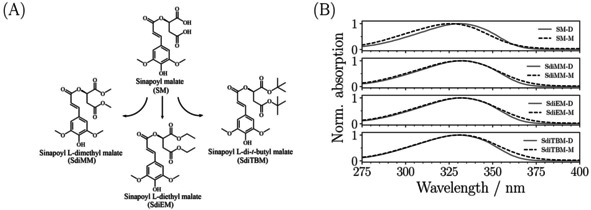
(A) The structures of the molecules studied in this work; sinapoyl malate (SM), sinapoyl L‐dimethyl malate (SdiMM), sinapoyl L‐diethyl malate (SdiEM) and sinapoyl L‐di‐*t*‐butyl malate (SdiTBM). (B) The UV‐visible spectrum of these molecules in dioxane (D; grey line) and methanol (M; dashed line).

Sinapoyl malate (SM; Figure 1(A)) has been identified as one such UV absorbing metabolite in *Arabidopsis thaliana* plants which is responsible (at least in part) for the plant's dynamic response to varying levels of UV exposure.[^6^, ^8^, ^9^, ^10^, ^11^, ^12^] Recently, a number of studies have been carried out focussing on the photophysical properties of SM, specifically, how solution‐phase SM photodeactivates after UV‐B photoexcitation.[^13^, ^14^, ^15^, ^16^] These point to SM (and similar derivatives[^17^, ^18^, ^19^, ^20^]) exhibiting a relaxation mechanism upon UV excitation involving ultrafast internal conversion from a photoexcited ππ* state to reform predominantly the original ground state *trans*‐isomer, occurring along a *trans‐cis* isomerisation reaction coordinate.

This relaxation mechanism, being mediated by the *trans‐cis* isomerisation coordinate, is particularly interesting since it is readily observed in the solution phase and involves ππ* dynamics, whereas analogous gas‐phase experiments involve significant nπ* dynamics and the different isomers are not readily distinguished.[^15^, ^17^, ^18^, ^19^, ^21^, ^22^] It is on this solution phase mechanism where this work focusses. We present transient absorption measurements of sinapoyl malate derivatives (Figure 1(A)) whose carboxyl groups are augmented to include increasingly large alkane chains to observe any changes to the *trans*‐*cis* mediated relaxation pathway. Specifically, we consider sinapoyl L‐dimethyl malate (SdiMM), sinapoyl L‐diethyl malate (SdiEM) and sinapoyl L‐di‐*t*‐butyl malate (SdiTBM) whose structures are shown in Figure 1. Rather strikingly, even in the case of *t*‐butyl groups, the largest steric augmentation studied in this work, all molecules appear to relax via the *trans*‐*cis* mediated pathways observed with the native sinapoyl malate molecule,[^14^, ^15^, ^17^] displaying a phenomenal level of conservation for this photodeactivation mechanism. Such conservation opens up the possibility to augment SM and its derivatives to improve desired photophysical properties and chemical compatibility within sunscreen mixtures, whilst retaining their underlying photodynamic properties.

## 2 Results and Discussion

**Figure 2:**
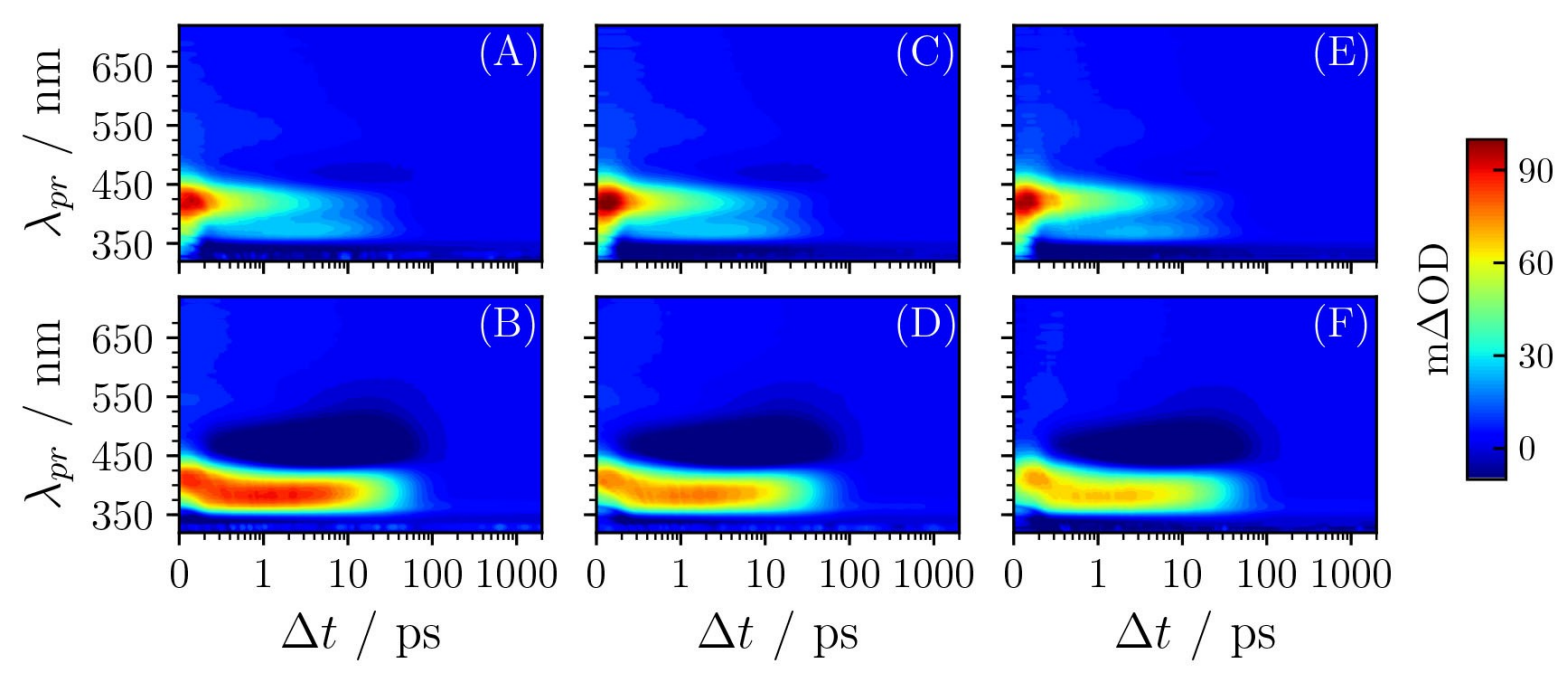
(A) and (B) TAS of SdiMM in dioxane and methanol respectively. (C) and (D) TAS of SdiEM in dioxane and methanol respectively. (E) and (F) TAS of SdiTBM in dioxane and methanol respectively. All TAS are have been chirp‐corrected for display here using the KOALA package [24].

We consider first the photoexcitation of SdiMM in dioxane. For SdiMM‐dioxane (Figure 2(A)) the TAS is dominated by three main features. The first feature is an intense absorption which is centred around probe wavelengths of ∼425 nm and decays away to the baseline by ∼30–50 ps. The second feature is a broad absorption which spans probe wavelengths between ∼425–650 nm, which decays away to the baseline by ∼2 ps. The third is a negative feature observed below ∼350 nm which persists to the maximum available delay time of 2 ns. The two positive features are readily attributed to excited state absorption (ESA), through comparison with previously studied molecules,[^13^, ^14^] originating from the molecule's 1^1^ ππ* electronic state (*i. e*. S_*n*_←1^1^ ππ*; *n*>1) which is prepared by the initial photoexcitation of the 330 nm pump‐pulse (1^1^ ππ*←S_0_, see SI). These features are in close agreement with previous measurements of SM.^[14]^ Through comparison with the UV‐visible spectrum of SdiMM (Figure 1(B)), the negative feature is assigned to a ground state bleach (GSB), which grows in for increasing pump‐probe delay times but does not fully recover by 2 ns. This incomplete recovery of the ground state is attributed to the production of a stable photoproduct, namely the *cis*‐isomer of SdiMM (*vide infra*). Moving now to SdiMM‐methanol, the same three features are observed although the central 1^1^ ππ* ESA is spectrally blue‐shifted, appearing centred around 375 nm. Furthermore, a strong negative feature is observed centred around 475 nm which is attributed to stimulated emission.[^13^, ^14^, ^23^] The TAS of SdiEM and SdiTBM both display similar features and are shown in Figure 2.

To understand the dynamical process occurring during photodeactivation, a sequential global fitting procedure is employed on the TAS (see SI for more details). The resulting lifetimes of the dynamical processes are summarised in Table 1, with Evolution Associated Difference Spectra (EADS) presented in the SI. We now rationalise the processes which are characterised by these lifetimes, making reference to previous work on SM.^[14]^ The similarity between all TAS for each respective solvent lead us to conclude that the process for photodeactivation remains largely unchanged upon increasing the steric complexity of these SM derivatives.

**Table 1:** Summary of the lifetimes of dynamical processes observed in sinapoyl malate (SM) (from previous work^[14]^) and its derivatives studied in this work: sinapoyl L‐dimethyl malate (SdiMM), sinapoyl L‐diethyl malate (SdiEM), sinapoyl L‐di‐*t*‐butyl malate (SdiTBM), in dioxane or methanol.

**SM**	Dioxane	Methanol
*τ*_1_/fs	119±40	619±101
*τ*_2_/ps	1.62±0.15	4.81±0.77
*τ*_3_/ps	22.4±1.9	33.5±1.7

**SdiMM**	Dioxane	Methanol
*τ*_1_/fs	205±40	603±40
*τ*_2_/ps	2.23±0.07	5.38±0.16
*τ*_3_/ps	27.6±0.8	33.6±1.0

**SdiEM**	Dioxane	Methanol
*τ*_1_/fs	215±40	600±40
*τ*_2_/ps	1.75±0.05	5.77±0.17
*τ*_3_/ps	26.1±0.8	33.7±1.0

**SdiTBM**	Dioxane	Methanol
*τ*_1_/fs	57±40	477±40
*τ*_2_/ps	1.23±0.04	4.37±0.14
*τ*_3_/ps	22.1±0.7	36.3±1.1

Beginning with the dioxane environment, we propose, as with SM,^[14]^ that the first lifetime, *τ*_1_, comprises a number of different mechanisms including including geometry rearrangement of the excited molecule and solvent reorganisation. This seems to occur in SdiTBM in around half the lifetime or shorter than the other SM derivatives. While the error associated with these short time constants (∼40 fs, which is limited by our instrument response) may reduce the relative differences between the derivatives, *τ*_1_ in SdiTBM is consistently shorter within said error than *τ*_1_ for SdiMM and SdiEM. This is likely due to the increased density of states within the larger molecule increasing the rate of *τ*_1_‐mechanisms, thus facilitating motion out of the Franck‐Condon region of the 1^1^ ππ* state.

**Figure 3:**
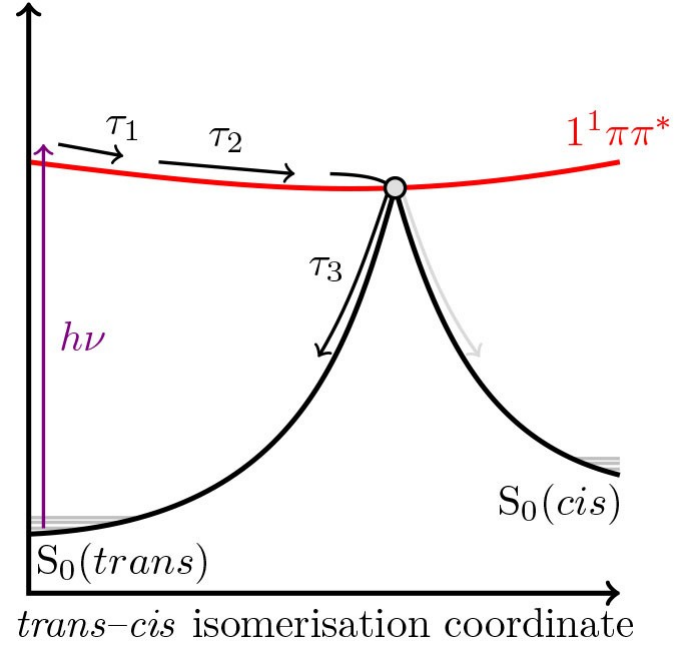
A schematic of the suggested photo deactivation mechanism observed in the molecules studied.

The process associated with the time constant *τ*_2_ is attributed to population evolving along the excited state potential energy surface. Previously for SM, two mechanisms were suggested which describe this motion.^[14]^ One mechanism attributes *τ*_2_ to motion from the 1^1^ ππ* state to a 2^1^ ππ* state, which are calculated as lying very close together in energy (see SI), that then couples to the ground state via a 2^1^ ππ*/S_0_ conical intersection (CI). The second mechanism involves motion along the 1^1^ ππ* only, which couples to the ground state via a 1^1^ ππ*/S_0_ CI, see Figure 3. Given the similarities between SM and the derivatives studied here, we suggest the same deactivation mechanism is present in all molecules studied here. Recent work by Zhao *et al*. helps to discriminate between these mechanisms, where they suggest that such a CI between the 1^1^ ππ* and 2^1^ ππ* state does not exist in SdiMM,^[20]^ but rather the latter mechanism is more plausible. Hence, we suggest that *τ*_2_ likely corresponds to motion along the 1^1^ ππ* state out of the Franck‐Condon region en route to the CI. The process *τ*_3_ is then attributed to the population flowing from the 1^1^ ππ* state to the ground state via a 1^1^ ππ* state/S_0_ CI, to reform the ground state. Some of the population completes isomerisation and persists as the long‐lived *cis*‐isomer photoproduct. The long‐lived final EADS is therefore assigned to the presence of the *cis*‐isomer stable photoproduct.

**Figure 4:**
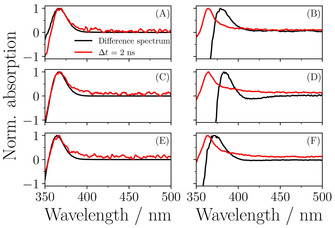
Continuous wave irradiation experiments of: SdiMM‐dioxane and SdiMM‐methanol (A) and (B) respectively; SdiEM‐dioxane and SdiEM‐methanol (C) and (D) respectively; SdiTBM‐dioxane and SdiTBM‐methanol (E) and (F) respectively. Black lines represent the difference spectrum for the irradiated sample displaying a clear absorption feature which appears centred around 370 nm after irradiation, attributed to the formation of the *cis*‐isomer. These resemble the feature observed in TEAS measurement of the samples at pump‐probe delay times (Δ*t*) of 2 ns, shown by the red lines.

The assignment of the final EADS to the *cis*‐isomer is supported by the difference spectra recorded in the steady state irradiation experiments (see Figure 4(A), (C), and (E)). These show, for all derivatives in dioxane, a close correlation between the difference spectra and the transient absorption spectra taken at Δ*t*=2 ns. This suggests that the photoproduct observed in the TAS is stable and long‐lived. Comparison with previous work on SM,^[14]^ which shows an identical long‐lived feature, confirms this to be due to the *cis*‐isomer.

Moving now to the methanol environment, we propose that the same mechanism is taking place here in all three SM derivatives, as was observed in the dioxane environment and again in SM.^[14]^ In methanol, the contribution to *τ*_1_ from geometry relaxation is clearly visible in a blue‐shift over time of the ESA observed around 400 nm. The longer lifetime of ∼400–600 fs makes this feature much more obvious in methanol than it was in dioxane and affirms our assignment for dioxane of this first lifetime including, at the very least, a contribution from vibrational energy redistribution coupled with geometric rearrangements. Likewise, *τ*_3_ reporting population flowing from the 1^1^ ππ* to S_0_ is evidenced through the stimulated emission seen in the TAS, which diminishes along a similar timeframe to the ESA.

Photodeactivation then proceeds through the same mechanism as suggested for the dioxane environment, finally resulting once more in a long‐lived photoproduct present in and close to identical between all three SM derivatives. The exact identification of this long‐lived feature is, however, more complicated in methanol than it was in dioxane.

To fully understand the nature of the long‐lived component in the TAS of the SM derivatives in methanol, we turn first to Figure 4(B), (D), and (F). From these data we can see that there is, in all cases, a red‐shift evident between the long‐lived TAS and the difference spectra, although this shift is only a few nm in SdiTBM, and is ∼20 nm in SdiMM and SdiEM. A similar shift of ∼20 nm was observed in previous studies on SM itself and attributed to the production of an unstable radical species observed (based on the fact that it was two‐photon dependant, see below, and matched well with known radical signals in similar systems) in the TAS but not in the difference spectrum.[^14^, ^25^, ^26^, ^27^, ^28^] This was confirmed by analysis of the power dependence of the long delay time TAS, which was found to be two‐photon dependent. It should also be noted that the NMR studies of SM post‐irradiation confirmed production of the *cis*‐isomer.^[15]^ We have undertaken equivalent power studies here, measuring the signal at the peak of the absorption feature in the 2.5 nm TAS, as well as the tail of this feature towards redder wavelengths, for all three SM derivatives in methanol. These were then plotted as a log‐log plot against excitation pulse power (see SI). We found that, in SdiMM and SdiEM, the peak of the 2.5 ns absorption (363 nm) had a two‐photon dependence whereas the tail (383 nm) showed a ∼1.4 photon dependence. This suggests that this TAS comprises two separate features which overlap to some extent; one with two‐photon dependence, likely a radical absorption; and one with one‐photon dependence attributed to *cis*‐isomer and triplet‐state formation.[^26^, ^29^, ^30^] While the signal from the *cis*‐isomer is masked by that of the radical feature, we are confident that it will contribute to the 2 ns TAS of all SM derivatives in methanol. This is due to the strong similarities between the lifetimes observed in methanol versus dioxane, and the positive identification of the *cis*‐isomer in SM[^14^, ^15^] both of which indicate that the primary one‐photon decay mechanism of SM and its derivatives is unchanged in both methanol and dioxane. In SdiTBM, the peak of the feature showed a 1.4 photon dependence, whereas the tail was very close to one‐photon, suggesting that the two‐photon signal is much less favoured in the more sterically hindered SM derivative.

Taking all of this evidence together, we postulate that in the cases of SM, SdiMM and SdiEM, a strong radical absorption is indeed present, which sits on top of a signal from triplet‐state formation and the *cis*‐isomer. In the case of SdiTBM, this radical feature is far less obvious. As the power dependence in all the SM derivatives in dioxane is one‐photon, and the long‐lived TAS and difference spectra in dioxane match, it is clear that no radical feature is present in the non‐polar solvent. There is, however, evidence of a weak triplet‐state signature (a tail in the ESA stretching towards lower energy; see Figure 4) similar to that seen in methanol. As the triplet‐state signal in both solvents appears weak, and given the difference spectra in Figure 4 (along with previous studies[^14^, ^15^]) implicate the creation of the *cis*‐isomer, we hypothesise that triplet‐state formation is out‐competed by *cis*‐isomer formation. As such, it is not considered a significant pathway in the one‐photon deactivation of these molecules. This leads us to conclude that the presence of the polar protic solvent is impacting the potential energy landscape of the molecules. Not only is interaction with the polar‐protic solvent clearly promoting radical formation (although the precise mechanism of this requires further investigation), but it also appears to shift the energy gap between the ground and first excited state of the *cis*‐isomer relative to that of the *trans*‐isomer, as evidenced by the red‐ shift observed in the difference spectra of the SM derivatives in methanol relative to those in dioxane. The increased steric hindrance between the methanol solvent and the ester groups of SdiTBM, caused by the addition of large *t*‐butyl groups to SdiTBM reduces the interactions between the solvent and the solute, and thus the radical species appears to be less favoured and the difference spectrum in methanol approaches that in dioxane. This steric hindrance occurs due to the fact that the minimum energy geometry of the ground state, causes the *t*‐butyl groups to point back in towards the first oxygen on the tail of SdiTBM, blocking the methanol from attacking this oxygen (see SI Figure S6). We suggest that one reason for the greater production of radicals in methanol solvent compared to dioxane may be due to the hydrogen bond between the first oxygen of the tail of the SM derivatives and the methanol solvent stabilising the quinoidal form of the phenoxy radical. This would explain why steric hindrance of this hydrogen bond in SdiTBM reduces the strength of the radical feature compared to the one‐photon signal observed. However, we are unable to rule out other possibilities (the sinapic acid radical would be one such other possibility) and while the two‐photon production of radicals in polar protic solvents is interesting, it has little impact on the role of these molecules as UV filters in nature, as the power density of UV light that they are likely to encounter will be far below those necessary for significant two‐photon absorption. Indeed, further work which incorporate implicit or explicit solvent models would be crucial to understand such solvent effects on radical absorption but is beyond the scope of the present work. More striking and of greater photoprotective relevance is that the large steric differences between SM and SdiTBM appears to have virtually no impact on the one‐photon deactivation mechanisms observed. This presents an interesting opportunity to augment such molecules to change their photophysical or chemical properties to better match the requirements of sunscreen mixtures, whilst preserving their photodynamics.

## 3 Conclusions

In the above work we studied the ultrafast photodynamics of three sinapoyl malate (SM) derivatives: sinapoyl L‐dimethyl malate (SdiMM), sinapoyl L‐diethyl malate (SdiEM) and sinapoyl L‐di‐*t*‐butyl malate (SdiTBM). We have demonstrated that the isomerisation of SM is preserved throughout major physical changes to the molecule,^[14]^ with the transient absorption spectra of all three derivatives in methanol and dioxane remaining remarkably similar to one another. In both solvents, upon photoexcitation, there is first an ultrafast <1 ps component corresponding to a variety of processes, including vibrational energy redistribution and geometric and solvent rearrangement around the excited molecule. A second process occurs in <10 ps in which the excited state population likely propagates along the 1^1^ ππ* state and couples to the ground state via a 1^1^ ππ*/S_0_ CI,[^14^, ^20^] although theoretical studies are needed to help understand the excited state dynamics of these molecules. Population then proceeds through the CI in <100 ps to repopulate the ground state via a *trans‐cis* isomerisation pathway. We have shown that the *cis*‐photoproduct is long‐lived in all cases. When dissolved in methanol, SdiMM and SdiEM show marked production of an unstable radical species, and a ∼20 nm shift in the difference spectrum after irradiation. For SdiTBM in methanol, radical formation is present but to a lesser extent and the shift observed in the difference spectrum is only a few nm. The incredible level of conservation of the isomerisation pathways in these SM derivatives demonstrates their importance as photoprotective molecules in not only plants, but also as candidates for tunable constituents in next generation sunscreen mixtures.

## Experimental and Computational Details

The experimental setup used has been described in detail elsewhere;^[31]^ the particulars of this experiment are given in brief here. A 1 mM solution of SdiMM in dioxane and methanol is photoexcited at its absorption maximum with 330 nm pump pulses (*cf*. Figure 1). The sample is recirculated inside a flow‐through cell (Harrick Scientific) between two CaF_2_ windows separated with 0.5 mm PTFE spacers. Probe pulses, generated from a white light continuum (∼320–700 nm), are used to investigate the photoexcited sample. Probe pulses are generated by focussing a small portion of the output of the 800 nm Ti:Sapphire fundamental (Spitfire; Spectral Physics) onto a 1 mm CaF_2_ crystal. Pump pulses are generated by a tunable optical parametric amplifier (TOPAS‐Prime; Light Conversion). Probe pulses are held at 54.7 ° relative to the pump‐pulses and are delayed after pump photoexcitation up to a maximum of 2 ns, through the use of a hollow gold retroreflector in the path of the probe beam.[^2^, ^31^] SdiEM and SdiTBM were investigated using an identical procedure. The collected transient absorption spectra (TAS) are chirp corrected and analysed through the use of a global fitting procedure provided in the Glotaran package,^[32]^ using a sequential kinetic model.

Continuous wave UV irradiation studies were performed on all samples using the following procedure. First, a steady‐state UV‐visible spectrum of the sample (typically a few micromolar concentration in dioxane or methanol) is recorded (Cary 60 spectrophotometer; Agilent), which is referred to as a *‘before*’ spectrum. The sample is then irradiated at 330 nm (±1 nm; ∼5 mW) for 10 minutes (Fluorolog3; Horiba). Following irradiation, another UV‐visible spectrum is taken referred to as the ‘*after*’ spectrum. Subtracting the before spectrum from the after spectrum results in the reported ‘*difference*’ spectrum analysed in this work.

*Ab initio* electronic structure calculations are employed to understand the excited states of the studied molecules.^[33]^ All *ab initio* calculations were performed within the package TURBOMOLE.[^34^, ^35^, ^36^, ^37^, ^38^] Ground state geometry optimisations were performed with Møller‐Plesset perturbation theory of order 2 (MP2) level of theory with the def2‐TZVPP basis set.[^39^, ^40^, ^41^] Ground state minimum geometries are confirmed through vibrational frequency calculations. Excited state calculations were performed at the Algebraic Diagrammatic Construction (ADC(2)) level of theory using the def2‐TZVPP basis set.[^42^, ^43^] Structural visualisation and analysis were performed within various software packages.[^44^, ^45^, ^46^, ^47^] More details can be found in the Supporting Information (SI).

## Conflict of interest

The authors declare no conflict of interest.
